# Self‐Powered Frequency‐Selective Acoustic Sensor Based on Bound States in the Continuum

**DOI:** 10.1002/advs.202410379

**Published:** 2025-02-21

**Authors:** Chao Song, Sibo Huang, Hongyu Ma, Shuhuan Xie, Din Ping Tsai, Jie Zhu, Yong Li

**Affiliations:** ^1^ Institute of Acoustics Tongji University Shanghai 200092 China; ^2^ Department of Electrical Engineering City University of Hong Kong Hong Kong 999077 China

**Keywords:** acoustic energy harvesting, acoustic sensor, bound states in the continuum, perfect absorption

## Abstract

Sound is a clean, renewable, and abundant energy source present ubiquitously in nature. However, it is often underutilized due to its low energy density in most environments. This study introduces a two‐state system that supports a Friedrich–Wintgen bound state in the continuum (BIC), achieving an unprecedented enhancement in sound energy density—up to 1849 times the incident sound intensity. By integrating this BIC‐supporting system with energy conversion mechanisms, such as piezoelectric films, high‐performance acoustic energy harvesting and sensing is realized. As a proof‐of‐concept, a self‐powered acoustic sensor system is developed. This sensor leverages the high‐quality‐factor nature of a BIC, providing exceptional passive frequency selectivity and the ability to activate a light‐emitting diode (LED) at the target frequency of 501 Hz with an offset of only 4 Hz. This work represents a groundbreaking advancement in sound‐energy enhancement, paving the way for BIC‐induced acoustic harvesters and sensors, with promising applications in wireless sensor networks and the Internet of Things.

## Graphical abstract

This innovative two‐state acoustic system leverages Friedrich–Wintgen bound states in the continuum to enhance local sound energy up to 1849 times the incident intensity, addressing the challenge of low energy density in acoustic energy harvesting. Its high quality factor and precise frequency selectivity demonstrate considerable potential to advance acoustic sensing and energy harvesting technologies.

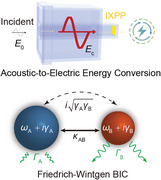

## 1 Introduction

Ambient energy is a desirable energy source due to its eco‐friendly and renewable merits. Acoustic energy is abundant and ubiquitous in both human habitats and natural environments. However, compared to other types of ambient energy such as solar, wind, and hydroelectric energy, acoustic energy is generally overlooked and wasted (usually regarded as unwanted noise) due to its relatively low energy density in most environments.^[^ ^1^, ^2^, ^3^ ^]^ Therefore, using artificial devices to enhance environmental acoustic energy density is a crucial technique for advancing energy harvesting and self‐powered sensing. Previous energy‐density‐enhancement devices for sound are mainly classified into three categories: Helmholtz resonators, Fabry–Perot resonators, and phononic crystals.^[^ ^3^, ^4^, ^5^, ^6^, ^7^, ^8^, ^9^, ^10^ ^]^ By combining these devices with energy‐conversion materials such as piezoelectric,^[^ ^11^, ^12^ ^]^ electromagnetic,^[^ ^13^ ^]^ and triboelectric^[^ ^14^, ^15^, ^16^ ^]^ materials, the power‐consuming thresholds of various applications can be fulfilled owing to the significantly amplified acoustic energy density. These energy‐density‐enhancement devices have achieved remarkable energy‐density amplification ranging from several tens of times to a reported maximum of 435.1 times.^[^ ^4^, ^11^, ^12^, ^14^, ^15^, ^16^, ^17^, ^18^ ^]^ However, the achievable level of energy‐density enhancement is still relatively low.

The effectiveness of an energy‐density enhancement resonant system is primarily governed by its quality factor (*Q*). A high *Q* not only boosts energy‐density amplification within the system but also enhances frequency selectivity, leading to substantial output power and exceptional sensitivity in sensing applications. However, achieving a high *Q* requires both a high radiative quality factor *Q*_rad_ and a high dissipative quality factor *Q*_loss_, which remains a challenge for normal resonators due to the fact that the pursuit higher *Q*_rad_ commonly accompanied with a lower *Q*_loss_.^[^ ^19^, ^20^, ^21^ ^]^ In acoustic systems, the intrinsic (thermal‐viscous) losses are closely associated with the degree of structural narrowness.^[^ ^22^, ^23^, ^24^ ^]^ Normal acoustic resonators such as Helmholtz resonators and Fabry–Perot resonators rely on reducing the connection areas between the resonators and the radiation field to achieve a low radiation loss (a high *Q*_rad_). Nevertheless, smaller connection areas inevitably result in narrower structure regions, giving rise to the increase of intrinsic loss (a lower *Q*_loss_). This inherent contradiction between *Q*_rad_ and *Q*_loss_ in normal acoustic resonant systems hinders the development of more powerful acoustic harvesters and more efficient self‐powered applications.^[^ ^25^, ^26^, ^27^, ^28^, ^29^ ^]^

In this study, we propose a two‐state system that supports a Friedrich–Wintgen bound state in the continuum (BIC), achieving unprecedented energy‐density enhancement and enabling self‐powered frequency‐selective sensing. BICs are characterized by their infinite high *Q*_rad_ and completely localized wave fields,^[^ ^21^, ^30^, ^31^, ^32^, ^33^, ^34^ ^]^ which have garnered considerable interest across various wave systems such as quantum,^[^ ^35^, ^36^, ^37^, ^38^ ^]^ optical,^[^ ^39^, ^40^, ^41^, ^42^, ^43^ ^]^ elastic,^[^ ^44^, ^45^, ^46^ ^]^ and acoustic systems.^[^ ^34^, ^47^, ^48^, ^49^, ^50^, ^51^, ^52^ ^]^ The proposed BIC‐supporting system can achieve a high *Q*_rad_ through the manipulation of resonance interactions without producing narrower structures of the system, offering new possibility for the realization of both a high *Q*_rad_ and a high *Q*_loss_.

## 2 Results and Discussion

### 2.1 Friedrich–Wintgen BIC for High‐Performance Acoustic Energy Harvesting

**Figure 1:**
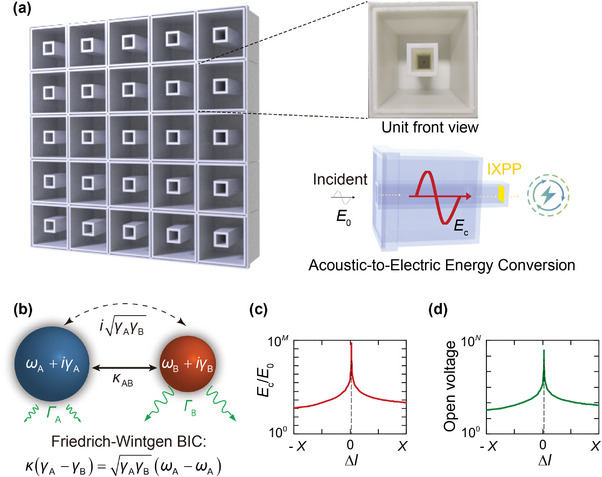
A BIC‐supporting system for local acoustic energy enhancement and improved harvesting. a) Schematic of the proposed two‐state system that supports a Friedrich–Wintgen BIC. The left figure is the structure array. The right top picture is the front view of the unit of the system (a fabricated sample). The right bottom figure illustrates the acoustic‐harvesting setup. *E*_0_ and *E*_c_ represent the energy density of the incident sound and the localized sound at the bottom of the nested cavity, respectively. b) Modulation of the fundamental modes of the two cavities of the two‐state system. c) Conceptual illustration of energy density enhancement with the modulation of the length difference between the center cavity and the surrounding cavity (Δ*l*). By adjusting Δ*l* within a specified range of length range (conceptually indicated from −*X* to *X*), the local energy enhancement can reach a high magnitude (conceptually indicated by 10^*M*^) near the BIC. d) Conceptual illustration of output open voltage of the proposed system with the modulation of Δ*l*. The output open voltage can reach a relatively high magnitude (conceptually indicated by 10^*N*^) near the BIC.

The unit cell of the proposed BIC‐supporting system consists of a center cavity nested within a surrounding wider cavity (**Figure** **1**a). The configuration is designed through an optimization process that aims to achieve stronger pressure field enhancement within the system (see Section S1.1, Supporting Information, for details). By modulating the length differences between the two cavities, the radiative quality factor (*Q*_rad_) of the system can be effectively adjusted from a relatively small value to infinity (corresponding to a Pure BIC). When the *Q*_rad_ is adjusted to close to the *Q*_loss_ of the system, significant enhancement energy density can be achieved at the bottom of the cavities, facilitating acoustic harvesting. The acoustic energy density can be expressed as $E=p_{a}^{2}/\left(2\rho_{0}c_{0}^{2}\right)$, where *p*_a_ is the pressure amplitude, ρ_0_ is the air density, *c*_0_ is the sound speed in the air. Employing the temporal coupled‐mode theory,^[^ ^53^ ^]^ the mode properties of the two coupled cavities (labeled as cavity A for the surrounding cavity and cavity B for the center cavity) can be theoretically analyzed (Figure 1b), where the corresponding mode functions (${\tilde{a}}_{A(B)}$) can be respectively expressed as^[^ ^53^, ^54^, ^55^ ^]^

(1) $$\begin{array}{ccc} \frac{d{\tilde{a}}_{A(B)}}{dt} & = & \left(i\omega_{A(B)}\right.-\left.\gamma_{A(B)}-\Gamma_{A(B)}\right){\tilde{a}}_{A(B)}+i\kappa {\tilde{a}}_{B(A)}\\ & & +\;i\sqrt{\gamma_{A(B)}}\left({\tilde{S}}_{i}^{+}+i\sqrt{\gamma_{B(A)}}{\tilde{a}}_{B(A)}\right) \end{array}$$

where ${\tilde{a}}_{A(B)}=a_{A(B)}e^{i\omega t}$, *ω* is the angular frequency, *ω*_A(B)_ is the angular resonant frequency of cavity A(B), *γ*_A(B)_ and Γ_A(B)_ indicate the radiation loss and the intrinsic loss of cavity A(B), respectively. *κ* and $\sqrt{\gamma_{A}\gamma_{B}}$ indicate the near‐field and the far‐field coupling among cavity A and cavity B, ${\tilde{S}}_{i}^{+}$ represents the incident waves. Accordingly, we have *Q*_radA(B)_ = *ω*_A(B)_/(2*γ*_A(B)_) and *Q*_lossA(B)_ = ω_A(B)_/(2Γ_A(B)_). Note that BICs are generally characterized by their radiative properties, where complete mode confinement results in zero radiation loss (*γ* = 0), independent of intrinsic loss and incident waves. Correspondingly, the Hamiltonian matrix of a proposed two‐state system can be obtained with ‐*i*∂*A*/∂*t* = *HA*, where $A={\left({\tilde{a}}_{A},{\tilde{a}}_{B}\right)}^{T}$

(2) $$H=\left(\begin{array}{cc} \omega_{A} & \kappa \\ \kappa & \omega_{B} \end{array}\right)+i\left(\begin{array}{cc} \gamma_{A} & \sqrt{\gamma_{A}\gamma_{B}}\\ \sqrt{\gamma_{A}\gamma_{B}} & \gamma_{B} \end{array}\right)$$

Based on Equation (2), the condition satisfying a Friedrich–Wintgen BIC can be expressed as^[^ ^56^ ^]^

(3) $$\kappa \left(\gamma_{A}-\gamma_{B}\right)=\sqrt{\gamma_{A}\gamma_{B}}\left(\omega_{A}-\omega_{B}\right)$$

The detailed eigenvalue analysis of the presented BIC‐supporting system and the mode pressure distributions are demonstrated in Section S1.2 and Figure S1 (Supporting Information). In the absence of intrinsic loss, the proposed two‐state system will have an infinite enhancement of energy density at the bottom of the nested cavity when the system approaches the Friedrich–Wintgen BIC, as conceptually illustrated in Figure 1c. This enhancement is fundamentally beneficial to acoustic‐to‐electric energy conversion. For instance, by attaching a piezoelectric film such as an irradiation cross‐linked polypropylene (IXPP), the theoretical output voltage can be predicted by^[^ ^11^, ^57^ ^]^

(4) $$\begin{array}{c} V_{m}=\frac{\omega_{n}RC_{p}d_{33}t_{p}/\varepsilon }{\sqrt{R^{2}C_{p}^{2}\omega_{n}^{2}\left(4\xi^{2}+{k_{p}}^{4}\right)+4\xi^{2}+4k^{2}\xi \omega_{n}RC_{p}}}\times \left(\frac{t_{p}{l_{p}}^{2}b_{p}}{6I}\right)\Delta p \end{array}$$

where Δ*p* is the pressure difference, *k*_p_ is the piezoelectric coupling coefficient, *C*_p_ is the piezoelectric capacitance, *R* is the loading resistance and *ε* is the permittivity, *b*_p_, *t*_p_, *l*_p_ is the width, the thickness, and the length of the piezoelectric material, respectively. *d*_33_ is the piezoelectric constant. ξ is the damping ratio. *I* is the moment of inertia of the whole film. Equation (4) demonstrates that the output voltage is proportional to the pressure amplitude of sound, where greater sound enhancement generally leads to larger output open voltage (Figure 1d). For other types of energy‐conversion materials such as triboelectric materials, similar energy‐correlations between the acoustic energy density and the output voltage also exist. For example, an empirical equation for a triboelectric film describes that *V*_m_ = 9.54Δ*p* + 0.13.^[^ ^14^ ^]^ Thus, energy‐density enhancement is a key technique to overcome the low‐energy‐density nature of sound and promote the development of various sound‐powered devices. In the following, we will demonstrate the advantage of the proposed BIC‐supporting system over traditional acoustic resonant systems in enhancing local energy density.

### 2.2 Local Energy Density Enhancement via a Two‐State BIC‐Supporting System

**Figure 2:**
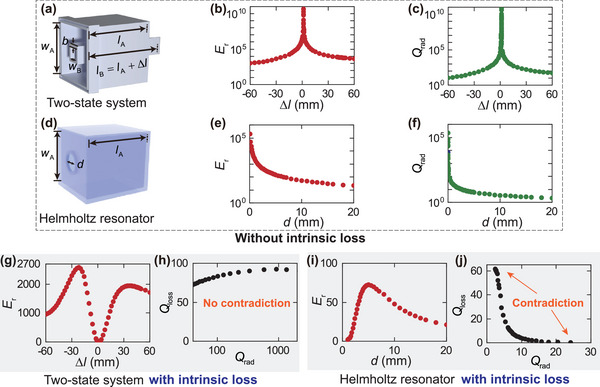
Lifting the contradiction between *Q*_rad_ and *Q*_loss_ via the two‐state BIC‐supporting system. a) Schematic of a two‐state system. The side lengths of cavity A (*w*_A_) and cavity B (*w*_B_) are set as 102 and 25 mm, respectively. The wall thickness *b* is 5 mm. The length of cavity A is fixed as *l*_A_ = 120 mm. b) Numerically calculated acoustic energy density enhancement factor, *E*_r_ with varying length differences (Δ*l*) between cavity A and cavity B, without considering intrinsic loss. c) *Q*_rad_ of the two‐state system. d) Schematic of a Helmholtz resonator. The width (*w*_A_) and length (*l*_A_) of its cavity are set to 102 mm and 120 mm, respectively. e) Numerically calculated *E*_r_ with varying diameters (*d*) of the Helmholtz resonator, without considering intrinsic loss. f) *Q*_rad_ of the Helmholtz resonator. g) Numerically calculated *E*_r_ of the two‐state system. h) *Q*_rad_ and *Q*_loss_ of the two‐state system for Δ*l* > 0 (Conditions of Δ*l* < 0 have a similar trend). i,j) Numerically calculated *E*_r_, *Q*_rad_, and *Q*_loss_ of the Helmholtz resonator.

To enhance the energy density of incident sound, numerous resonant structures and phononic crystals are presented.^[^ ^3^, ^4^, ^5^ ^]^ However, these traditional designs encounter the contradiction between low radiation loss and low intrinsic loss, limiting their achievable quality factors and the level of local energy density enhancement. In this work, our proposed two‐state BIC‐supporting system can realize a high *Q*_rad_ while maintaining a high *Q*_loss_, opening a new avenue for developing high‐*Q* acoustic devices for sound enhancement. To demonstrate the capability and advantage of the two‐state system, we compare the two‐state system and a Helmholtz resonator with similar external dimensions. First considering the situation without intrinsic loss, as shown in **Figure** **2**, we can find that the two‐state system can induce strong local energy density enhancement (more than 10^10^ with a limited frequency resolution) in the vicinity of the BIC Δ*l* = *l*_B_ − *l*_A_ = 1.341 mm (Figure 2a–c), and the Helmholtz resonator can also achieve great energy density enhancement when its neck diameter approaches zero (Figure 2d–f). The experimental verification of the BICs is demonstrated in Figure S2 (Supporting Information). In addition, the formation mechanism of the achieved BIC is explained based on the eigenvalue analysis in Section S1.4 (Supporting Information), demonstrating that this BIC is achieved by the interaction between the two modes of cavity A and cavity B. Here the acoustic energy density enhancement factor, *E*_r_, is defined as the ratio of energy density between the incident sound and the maximum localized sound within the resonant systems. Note that the results depicted in Figure 2b are calculated for the steady‐state acoustic field under a continuous incident wave excitation, where “Frequency Domain” in the preset “Pressure Acoustics” was chosen and no intrinsic loss was introduced in the simulations. The enhancement of the acoustic field within the BIC‐supporting system is achieved by confining the incident waves and accumulating the acoustic energy over time. In a hypothetical simulation setup without intrinsic loss, the accumulated energy in the system would keep increasing to infinite (as shown in Figure 2b). However, in practical applications where the intrinsic loss is inevitable, the maximum magnitude of the field enhancement is limited.

However, for the Helmholtz resonator, the small neck diameter for a high *Q*_rad_ results in narrow regions accompanied by substantial intrinsic loss, which significantly limits the *Q*_loss_ factor and reduces the magnitude of sound enhancement for practical structures with intrinsic loss. Conversely, the two‐state system realizes a high *Q*_rad_ through the resonance interaction between the two cavities, instead of relying on reducing the areas between the system, thereby enabling a higher *Q*_loss_. This advantage enables the two‐state system to induce much stronger local energy density enhancement than the Helmholtz resonator in the presence of intrinsic loss. As shown in Figure 2g,i, the maximum achievable *E*_r_ of the two‐state system is higher than two orders of magnitude than the Helmholtz resonator, which is fundamentally attributed to the lift of the contradiction between the *Q*_rad_ and *Q*_loss_ (Figure 2h,j). Note that the acoustically rigid side walls of impedance tubes can create a series of mirror images of the unit cell, which is equivalent to an infinitely repeated arrangement of the unit cell. Considering the subwavelength feature of the unit cell, the difference between rigid and periodic boundary conditions is very tiny for normal incidence. We have also performed simulations with periodic boundary conditions in Figures S3 and S4 (Supporting Information). The results for periodic boundary conditions and impedance tube conditions are almost identical. Previous study demonstrates that using combinations of multiple units of resonant structures in free field conditions can achieve effects similar to those observed in impedance‐tube systems with a unit cell.^[^ ^11^ ^]^

**Figure 3:**
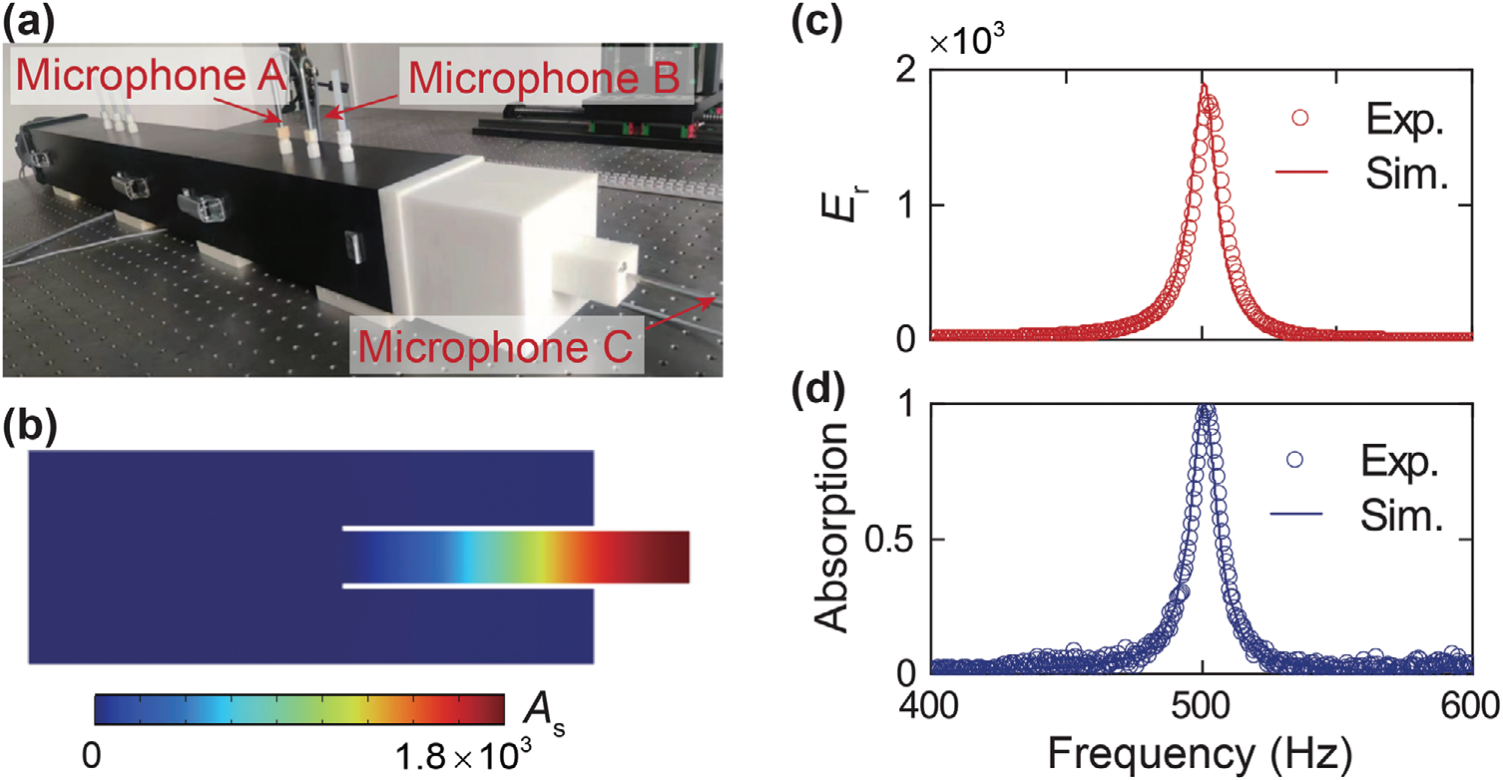
Acoustic energy density enhancement and perfect absorption. a) Experimental setup diagram for measuring the acoustic energy density inside cavity B and the absorption coefficients. b) Simulated distribution field of sound pressure (indicated by colors) inside the two‐cavity system and the radiation field at 501 Hz. The amplitude of the incident sound is 1 Pa. c) Acoustic energy density enhancement factors (*E*_r_) at the bottom of cavity B. d) Acoustic absorption coefficients of the two‐state system.

To validate the aforementioned theoretical and simulation results, we conduct experiments in an impedance tube. From Figure 2g, there are two peaks of *E*_r_ for Δ*l* < 0 and Δ*l* > 0, respectively. Considering the more robust qualities in sound enhancement and sound absorption (Figure S5, Supporting Information), we choose the Δ*l* = 45.75 mm to demonstrate our concept experimentally. The experimental setup is demonstrated in **Figure** **3**a, where the experimental sample is mounted at the end of the impedance tube. With this setup, the sound emitted from the loudspeaker will be effectively confined within the presented structure, leading to a large sound‐pressure amplification at the bottom of the center cavity (Cavity B), as illustrated by the simulated pressure field distribution in Figure 3b. The *E*_r_ at the bottom center of Cavity B are measured, which manifests in good accordance with the simulated results and realizes a peak value of 1849 at 501 Hz (Figure 3c). We also measured the sound absorption coefficients of the presented structure, which achieves a nearly perfect absorption coefficient exceeding 0.99 (Figure 3d). In most scenarios, the ambient sound energy is taken as unwanted noise, and the two‐state system can also serve as an efficient sound absorber for noise reduction. The absorption coefficient is related to the ratio of the incident wave area to the area of the sound‐absorbing structure. In practical applications where the impedance tube is removed, we can employ more periods of the actual structure to achieve satisfactory absorption performance, as illustrated in Figure 1. These results demonstrate that the presented quasiBIC‐supporting structure can efficiently trap the incident sound and induce a greatly enhanced local energy density compared to the incident sound waves.

### 2.3 Acoustic Energy Harvesting

**Figure 4:**
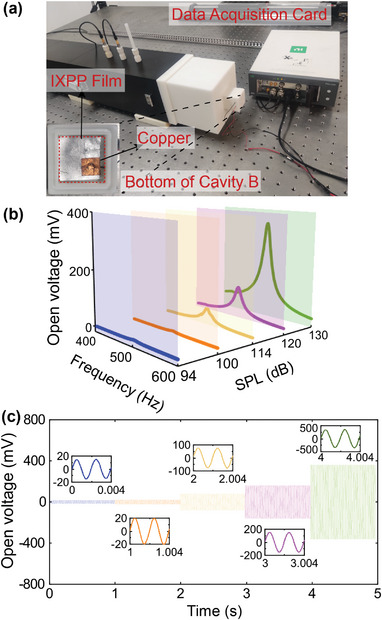
Acoustic energy harvesting. a) Schematic of the experimental setup for measuring the open voltage. b) Experimental results of the open‐circuit voltage of the system at incident sound pressure levels of 94–130 dB. c) Time‐domain waveforms of the open‐circuit voltage at the resonant frequency for different sound pressure levels in the system. The embedded images show detailed graphs of the open voltage corresponding to each sound pressure level.

Extreme sound confinement leads to a significant increase in sound energy density, which effectively facilitates acoustic energy harvesting. To achieve acoustic‐electrical energy conversion, IXPP piezoelectric film is attached to the bottom of cavity B. The area of the film area matches the cross‐sectional area of cavity B, as shown in the inset picture of **Figure** **4**a. The IXPP film is attached to the bottom of cavity B using double‐sided conductive adhesive. The wires are secured to the IXPP film using copper foil tape. To ensure the IXPP film is functioning correctly, we measure the output signals. A stable sine wave output indicates that the IXPP film is operating normally. The overall experimental setup for the acoustic‐harvesting system is illustrated in Figure 4a. The piezoelectric film is connected to a data acquisition card, and then the open‐circuit voltage resulting from the structure's interaction with incident sound pressure can be measured. The experimental results demonstrate that the open‐circuit voltage increases with the increasing amplitude of incident sound, where the peak open‐circuit voltage reaches 350 mV at 501 Hz for incident sound with a pressure level of 130 dB, as shown in Figure 4b. The open‐circuit voltage frequency response closely matches the cavity sound intensity amplification factor, indicating that the confinement of sound energy within the structure significantly enhances acoustic‐electrical energy conversion (see Section S1.7, Supporting Information).

Furthermore, we measure the transient open‐circuit voltages of the designed structure at its resonant frequency under different incident sound pressure levels, with the acquisition of corresponding time‐domain waveforms (Figure 4c). The close‐up views in the inset figure provide detailed information at the beginning of the pressure‐level transitions, which demonstrate that the output voltages are stable sinusoidal signals. The open‐circuit voltages are related to both the sound intensity amplification factor of the resonant structures and the properties of the acoustic‐electric interaction materials. Therefore, it is noteworthy that the open‐circuit voltages of the presented quasiBIC‐supporting system can be further improved by utilizing more efficient energy‐conversion materials.

### 2.4 Self‐Powered Acoustic Sensor

**Figure 5:**
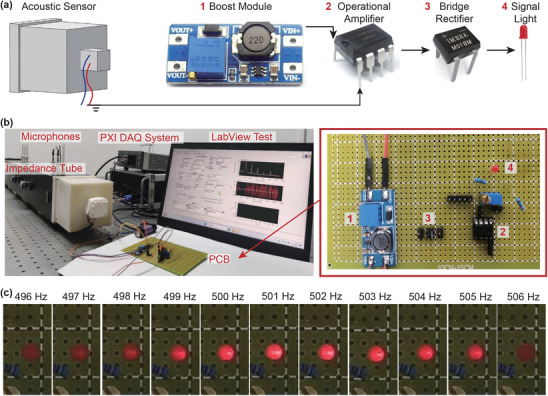
Self‐powered frequency‐selective acoustic sensor. a) Schematic diagram of the acoustic sensor system. b) A photograph of the experimental setup diagram of the acoustic sensor. The illustration is a detailed diagram of the circuit board. The labels in the diagram are consistent with that in Figure 5a. c) The experimental diagrams of the LED variations on the printed circuit board (PCB) with changing sound wave frequency.

Self‐powered acoustic sensors exhibit significant application potential in a wide range of emerging fields, including artificial cochlear implants,^[^ ^58^ ^]^ railway self‐powered monitoring systems,^[^ ^11^ ^]^ and the Internet of Things^[^ ^3^ ^]^ (see more detailed discussions on the future applications in Section S1.8, Supporting Information). The sharp resonance curve of the quasiBIC provides the possibility for developing frequency‐selective sensors. The frequency‐selectivity capability can spare the additional acoustic filtering systems,^[^ ^27^, ^59^ ^]^ enable accurate recognization of specific frequency characteristics,^[^ ^60^ ^]^ and benefit the modulation of frequency bandwidths for specific applications.^[^ ^61^ ^]^ For a quasiBIC‐empowered acoustic sensor, small frequency variations can induce significantly different responses, thereby rendering efficient distinctions between different frequencies in the frequency‐sensitive implementations. To construct such a high‐performance sensor, we build up the circuit board in the aforementioned harvester design to a boost module (MT 3608), an operational amplifier (LM 3558), a bridge rectifier (MB 10F), and a signal light, as shown in **Figure** **5**a. The boost module is for the power supply, with the output of the designed signal source connected to the operational amplifier. The input AC signal passes through the rectifier bridge, converting it into a DC source. The on/off state of the signal light can be controlled by the output of the acoustic sensor, which can validate the basic functionality of the acoustic sensor. In the experiments, sound wave signals at different frequencies (490–510 Hz) with the same sound pressure level are controlled by the LabView test panel, as shown in Figure 5b.

Figure 5c illustrates varying conditions of the light‐emitting diode (LED) on the circuit board under input sound waves within 496–506 Hz. A detailed experimental process is shown in Movie S1 (Supporting Information). As the frequency of the sound wave increases, we can observe the clear brightness variations of the LED, where the highest brightness can be found at the target frequency of 501 Hz (Figure 5c). The signal light can be only illuminated within the frequency range of 497–505 Hz, indicating the excellent frequency‐selectivity capability of the presented acoustic sensor. At 130 dB and 501 Hz, the measured electric power driving the loudspeaker is 968 mW, the acoustic power emitted from the loudspeaker is 50 mW, the significantly enhanced local acoustic power on the IXPP is 5149 mW, and the electric power used to light the LED is 4.3 mW. More detailed information on the measured power at different frequencies and under different sound pressure levels is demonstrated in Figure S7 (Supporting Information). Additionally, the scenario of white noise incidence is discussed in Section S1.10 (Supporting Information), where this self‐powered sensor can be used to detect thresholds of white noise. In fact, the presented system can offer superior frequency‐selective performances with smaller allowable frequency offsets by using signal lights with higher driving voltage sensitivity. In our current experiment, the driving voltage of the employed LED is in a relatively robust range of 1.8–2V, which enlarges the allowable frequency offset of the presented sensor.

## 3 Conclusion

In conclusion, we present an acoustic quasiBIC‐based system for a multifunctional device capable of enhanced‐intensity energy harvesting and high‐*Q* sensing. This high‐performance acoustic sensor and energy harvester not only offers precise and effective sensing and harvesting of sound waves at specific frequencies but also excels in sound absorption, sound field enhancement, and sound‐to‐electrical energy conversion. As a proof‐of‐concept design, we design a structure that experimentally achieves a high *Q* factor of 38.6 and considerable sound intensity enhancement within the system of 1849 times the incident sound intensity. The *Q* factor is calculated based on Figure 3d by dividing the resonant frequency by the full width at half‐maximum of the absorption coefficients. Furthermore, by integrating piezoelectric films and the presented quasiBIC‐supporting system, the harvested acoustic energy can be applied to drive the designed circuit, resulting in a voltage output of 350 mV under the incident sound of 130 dB. This integration system facilitates precise control over circuit operations based on the frequency of the sound waves, leading to an acoustic sensor with exceptional frequency‐selectivity capability. Such a quasiBIC‐based sensor system allows for precise customization of the target frequency as well as the spectral characteristic, making it versatile and suitable for frequency‐specific applications. Our work introduces the concept of acoustic quasiBIC‐based energy harvesting and sensing, which enriches the application platform of acoustic BICs and opens up a new avenue for the development of novel and high‐*Q* acoustic energy harvesters and self‐powered sensors.

## 4 Experimental Section

### Numerical Simulation

The function of the designed structure was validated through finite element analysis by using COMSOL Multiphysics. The mass density and sound velocity of the air‐filled domain were set to 1.21  kg m^−3^ and 343  m s^−1^, respectively. The boundaries of the designed structure and the impedance tube for the transmission were modeled as acoustically rigid materials since the acoustic impedance of the manufacturing materials of the samples and the impedance tube are considerably higher than that of air. The region of cavity B was set as “Narrow Region Acoustics.” The boundaries of cavity A were set as “Thermoviscous Boundary Layer Impedance.” Both models incorporate considerations for thermal conduction losses and viscous losses, and they do not require manual setting of boundary layers. However, the former was more applicable to regular acoustic structures (such as Cavity B with a rectangular cross‐section), while the latter was better suited to irregular acoustic structures (such as Cavity A). A probe at the bottom of cavity B acquires the pressure data.

### Sample Preparation and Experimental Procedures

The designed structures were fabricated using 3D‐printed resin material with a manufacturing precision of 0.1 mm. A steel acoustic impedance tube with a side length of 102 mm and a wall thickness of 8 mm was utilized to measure the reflection coefficients and absorption coefficients. A LabView program and a data acquisition card were employed to capture electrical signals from the piezoelectric film. A loudspeaker was used to generate a white noise signal at the opening of the impedance tube. The experimental samples are placed on the end (opposite to the opening) of the impedance tube. Two microphones with a 1/4‐inch diameter (Brüel & Kjær type‐4187), referred to as Microphone A and Microphone B, were employed to measure the magnitudes and phases of the sound waves at designated locations. Another microphone with a 1/8‐inch diameter (Brüel & Kjær type‐4137), labeled as Microphone C, was used to measure the pressure amplitudes and phases at the bottom of cavity B, as shown in Figure 3a. In the experimental setup for acoustic‐to‐electric conversion, the lead‐out wires were connected to the electrodes of the piezoelectric film. The film is affixed to the bottom of cavity B using conductive adhesive tapes.

## Conflict of Interest

The authors declare no conflict of interest.

## Supporting information

Supporting Information

Supplemental Movie

## Data Availability

The data that support the findings of this study are available from the corresponding author upon reasonable request.
