# Supplementary material for: Self‐Powered Frequency‐Selective Acoustic Sensor Based on Bound States in the Continuum
Source: Adv Sci (Weinh). 2025 Feb 21;12(15):2410379. doi: 10.1002/advs.202410379 (PMC12005764; doi:10.1002/advs.202410379)
Supplement: Supplementary file 1 — Supporting Information [file ADVS-12-2410379-s002.pdf]

ADVANCED SCIENCE
Open Access

Supporting Information

for *Adv. Sci.*, DOI 10.1002/advs.202410379

Self-Powered Frequency-Selective Acoustic Sensor Based on Bound States in the Continuum

Chao Song, Sibo Huang, Hongyu Ma, Shuhuan Xie, Din Ping Tsai, Jie Zhu* and Yong Li**

Supplemental Material for "Self-powered frequency-selective acoustic sensor based on bound states in the continuum"

Chao Song[1,†],Sibo Huang[2,†],Hongyu Ma[1],Shuhuan Xie[1],

Din Ping Tsai[2*],Jie Zhu[1*],Yong Li[1*]

[1]Institute of Acoustics, Tongji University, Shanghai 200092, China

[2]Department of Electrical Engineering, City University of Hong Kong, Hong Kong 999077, China

*dptsai@cityu.edu.hk *jie@tongji.edu.cn *yongli@tongji.edu.cn

†These authors contributed equally to this work.

The PDF file includes:

Supplementary Text

Figure S1 to S7

Movie S1

Supplementary Text

1.1 The design concept and process of the BIC-supporting system

The design concept of our proposed BIC-supporting system is based on the following principles:

1. We aim to leverage the high quality factors and strong energy localization of BICs to maximize the local acoustic energy density within the structure.

2. We employ a coupled system consisting of two cavities to form a Friedrich-Wintgen BIC.

3. In practical scenarios with intrinsic losses, our design utilizes a large cavity combined with a smaller cavity. This configuration intensifies the acoustic energy within the smaller cavity, which demonstrates superior performance in achieving large local acoustic energy density over the designs with two cavities of an equal cross-sectional area.

4. However, as the cross-sectional area of the smaller cavity is reduced, the intrinsic loss increases significantly, which in turn reduces the field enhancement. Therefore, we optimized the maximum achievable local acoustic energy density enhancement factor (E_{r}) by tuning the size of the smaller cavity while maintaining the larger cavity's cross-section equal to the cross-section of the impedance tube. This optimization yields an optimized cross-section of the smaller cavity.

Supplementary Text

1.2 Eigenvalue analysis of the presented BIC-supporting system and the mode pressure distributions

We have numerically calculated the eigenmodes of the coupled two-state system at the BIC (Δl = 1.341 mm) as follows: $\sigma_-/2\pi$ = 667.9 Hz (corresponding to the BIC) and $\sigma_+/2\pi$ = 661.53 + 272.7i Hz (corresponding to a lossy mode). Besides, for the individual component resonators, we can obtain their fundamental eigenvalues as $(\omega_{\mathrm{A}} + i\gamma_{\mathrm{A}})/2\pi$ = 661.58 + 251.43i Hz, $(\omega_{\mathrm{B}}+i\gamma_{\mathrm{B}})/2\pi$ =666.82 + 25.26i Hz. Then, Equation (3) can be satisfied when $\kappa/2\pi$ = - 1.92 Hz. To verify the derived value of κ, we can theoretically calculate the eigenmodes of the coupled two-state system by substituting this κ and the eigenvalues of the individual resonators into (referred to Refs. 51 and 56): $\sigma_- = \frac{\left(\omega_{\mathrm{A}}\gamma_{\mathrm{B}}+\omega_{\mathrm{B}}\gamma_{\mathrm{A}}-2\kappa\sqrt{\gamma_{\mathrm{A}}\gamma_{\mathrm{B}}}\right)}{(\gamma_{\mathrm{A}}+\gamma_{\mathrm{B}})}$, $\sigma_+ =$

$(\omega_A + \omega_B) - \frac{(\omega_A\gamma_B + \omega_B\gamma_A - 2\kappa\sqrt{\gamma_A\gamma_B})}{(\gamma_A+\gamma_B)}$. As a result, we have $\sigma_-/2\pi = 667.41$ Hz and $\sigma_+/2\pi = 661 + 276.69i$ Hz. The good agreement between the theoretically and numerically calculated eigenvalues validates the derived value of κ. The slight deviations observed between the simulation results and theoretical results may be attributed to the discrepancies between the idealized models used in the coupled-mode theory and the actual acoustical systems. In addition, the mode pressure distribution of the coupled two-state system at the BIC is shown in Figure S1 below, where the pressure distribution is confined within the system without radiation to the far field.

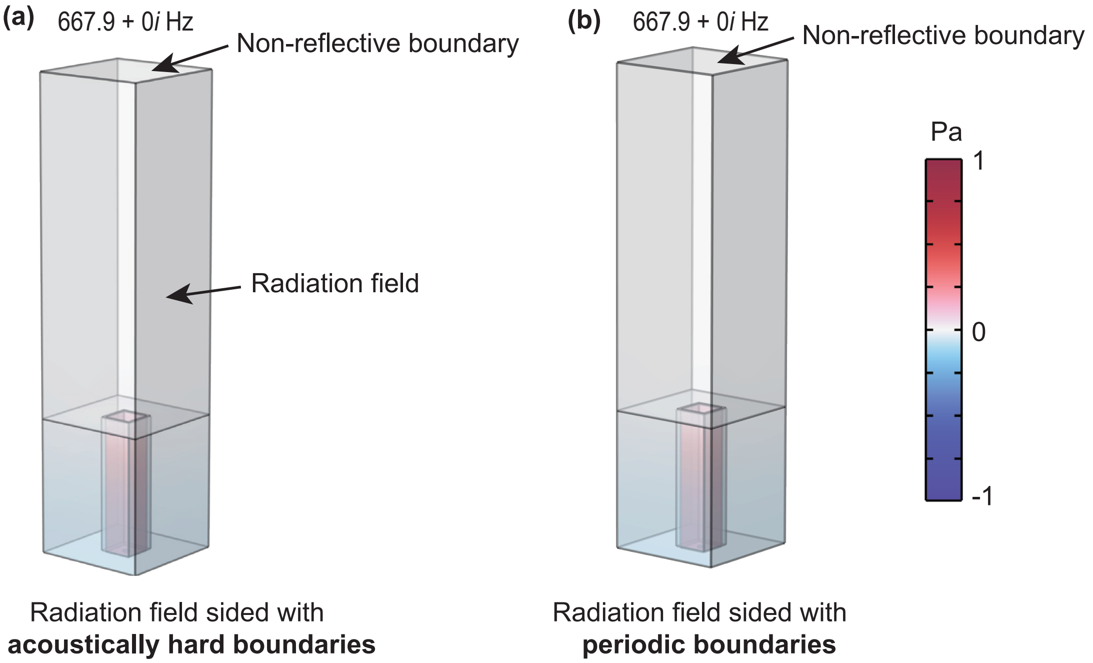

Figure S1. Mode pressure distributions of the BIC-supporting system at BICs. a) When the side walls of the radiation field are set as acoustically rigid boundaries and $\Delta l = 1.341$ mm, the presented two-state system supports a pure BIC with a purely real eigenvalue. b) When the side walls of the radiation field are set as periodic boundaries and $\Delta l = 1.341$ mm, the presented two-state system also supports a pure BIC with the same purely real eigenvalue. The pressure distributions are normalized. The remaining geometry parameters of the BIC-supporting system here are the same as those in Figure 2a.

Supplementary Text

1.3 Experimental demonstration of the existence of the BIC

The reflection coefficient diagram of the coupled system with the varying lengths of cavity B is calculated based on numerical simulations, where the length of cavity A is fixed as 120 mm (Figure S1a). When the two cavities reach a certain length difference (Δl), a vanishing linewidth, marked by the white pentagon (1.341, 665.423), can be observed, which indicates an infinite Q factor. Figure S1b demonstrates the reflection spectra of the presented system at three specific values of Δl, illustrating the system's reflection qualities when crossing the condition of the BIC. When $\Delta l = 9$ mm, the system supports a quasiBIC, which leads to a reflection valley. Then, when $\Delta l = 1.341$ mm, the quasiBIC turns into a pure BIC, and the reflection valley is missing due to the complete isolation feature of the BIC. Furthermore, when Δl deviates from 1.341 mm, the BIC will turn into a quasiBIC. As a result, a reflection valley will appear. This manifests the extraordinary potential of the quasiBIC-supporting system for achieving greatly enhanced sound-intensity fields.

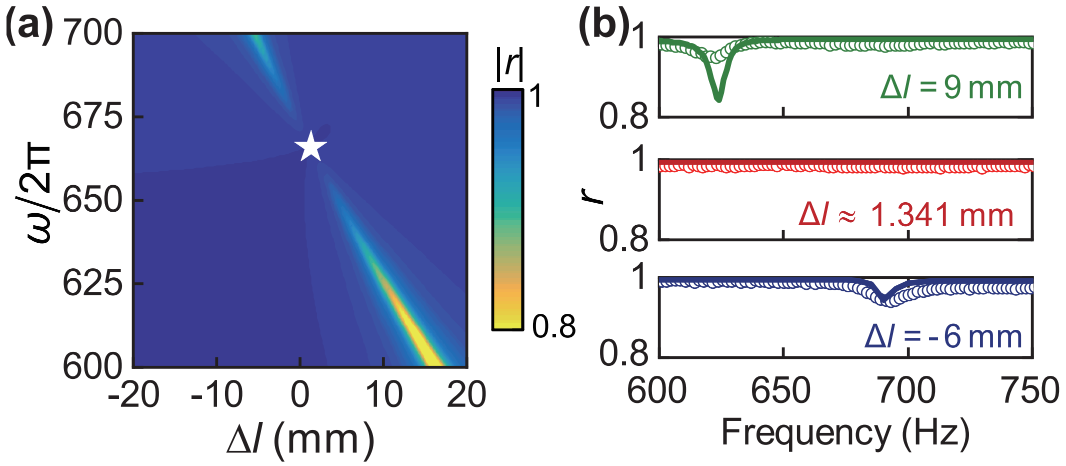

Figure S2. Demonstrations of the existence of the BIC and the quasi-BIC-induced field enhancement. a) Numerically calculated reflection amplitude as a function of the cavities' length difference Δl and frequency, $\omega/2\pi$. $\omega_A = 1.05\gamma_A$, $\omega_B = 0.038\gamma_B$. b) Measured (circles) and simulated (solid lines) reflection spectra for designed structures with different cavity lengths Δl. The cavity depth differences from the top panel to the bottom panel are as follows: 9 mm, 1.341 mm, and -6 mm, respectively.

Supplementary Text

1.4 The formation mechanism of the achieved BIC

For the coupled system consisting of cavity A and cavity B, the eigenvalues of the Hamiltonian matrix of the coupled system can be expressed as $\sigma_- = \omega_{\text{ave}} + i\gamma_{\text{ave}} - \sqrt{(\kappa+\mu)^2 + (\omega_{\text{diff}} + i\gamma_{\text{diff}})^2}$ and $\sigma_+ = \omega_{\text{ave}} + i\gamma_{\text{ave}} + \sqrt{(\kappa+\mu)^2 + (\omega_{\text{diff}} + i\gamma_{\text{diff}})^2}$, where $\omega_{\text{ave}} = (\omega_{\text{A}} + \omega_{\text{B}})/2$, $\gamma_{\text{ave}} = (\gamma_{\text{A}} + \gamma_{\text{B}})/2$, $\omega_{\text{diff}} = (\omega_{\text{A}} - \omega_{\text{B}})/2$, $\gamma_{\text{diff}} = (\gamma_{\text{A}} - \gamma_{\text{B}})/2$, and $\mu = i\sqrt{\gamma_{\text{A}}\gamma_{\text{B}}}$. The eigenvalues of the coupled system arise from the interference between the modes of cavity A and cavity B. This coupling mechanism of the two modes of cavity A and cavity B is interpreted in the two eigenvalues. By calculating the resonant frequencies and radiation losses of cavity A ($(\omega_{\text{A}} + i\gamma_{\text{A}})/2\pi$ = 661.58 + 251.43i Hz) and cavity B ($(\omega_{\text{A}} + i\gamma_{\text{A}})/2\pi$ = 666.82 + 25.26i Hz) and substituting them into the eigenvalues of the coupled system (derived $\kappa/2\pi$ = - 1.92 Hz), we can obtain that $\sigma_-/2\pi$ = 667.41 Hz and $\sigma_+/2\pi$ = 661 + 276.69i Hz. The purely zero eigenvalue, σ_-, corresponds to the BICs, which is proved to be achieved based on the interaction of the modes of cavity A and cavity B. And σ_+ indicates a lossy mode, which is also a typical feature accompanied by a Friedrich-Wintgen BIC.

Supplementary Text

1.5 The presented BIC-supporting system with periodic boundary conditions

In this work, we experimentally investigate an isolated component containing two resonators within an impedance tube whose side walls can be considered acoustically rigid. Actually, the acoustically rigid side walls can create a series of mirror images of the unit cell, which is equivalent to an infinitely repeated arrangement of the unit cell. Therefore, as shown in Figure S3 and S4, the results obtained under periodic boundary conditions and in impedance tube tests are nearly identical, both in the absence and presence of intrinsic loss.

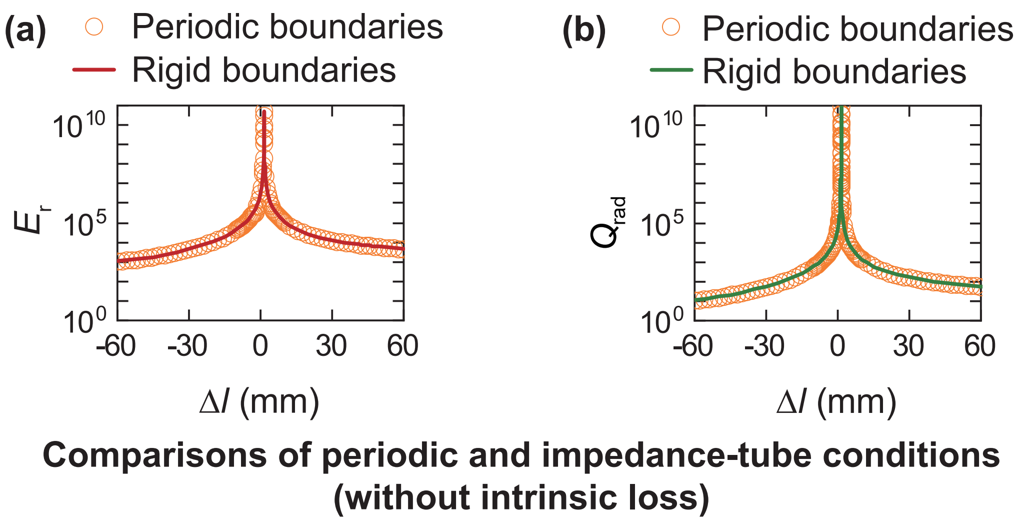

Figure S3. The presented BIC-supporting system with periodic and acoustically-rigid boundaries without intrinsic loss. a) Numerically calculated acoustic energy density enhancement factor (E_r). b) Qrad of the two-state system. The geometry parameters of the BIC-supporting system here are the same as those in Figure 2b-c.

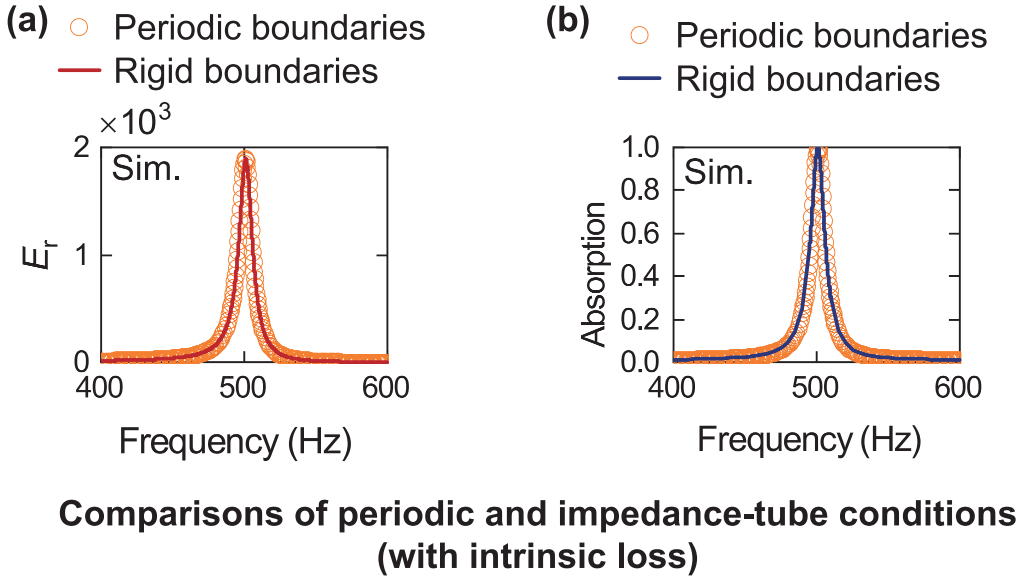

Figure S4. The presented BIC-supporting system with periodic and acoustically-rigid boundaries with intrinsic loss. a) Numerically calculated acoustic energy density enhancement factor (E_r). b) Absorption coefficients of the two-state system. The geometry parameters of the BIC-supporting system here are the same as those in Figure 3c-d.

Supplementary Text

1.6 Acoustic absorptions of the two-state system and the Helmholtz resonator

The presented system can achieve sound confinement (perfect absorption) of incident acoustic waves when the radiation loss of its supporting QBIC equals the intrinsic loss of the practical system. Figure S5 illustrates the energy density enhancement and peak absorption coefficients of the presented two-state system and the Helmholtz resonator. The structures exhibiting good energy density enhancement also show excellent sound absorption properties. Comparing the conditions of Er with different cavity lengths $\Delta\ l < 0$ and $\Delta\ l > 0$, the latter condition shows greater robustness of both energy density enhancement and absorption to the change of length difference.

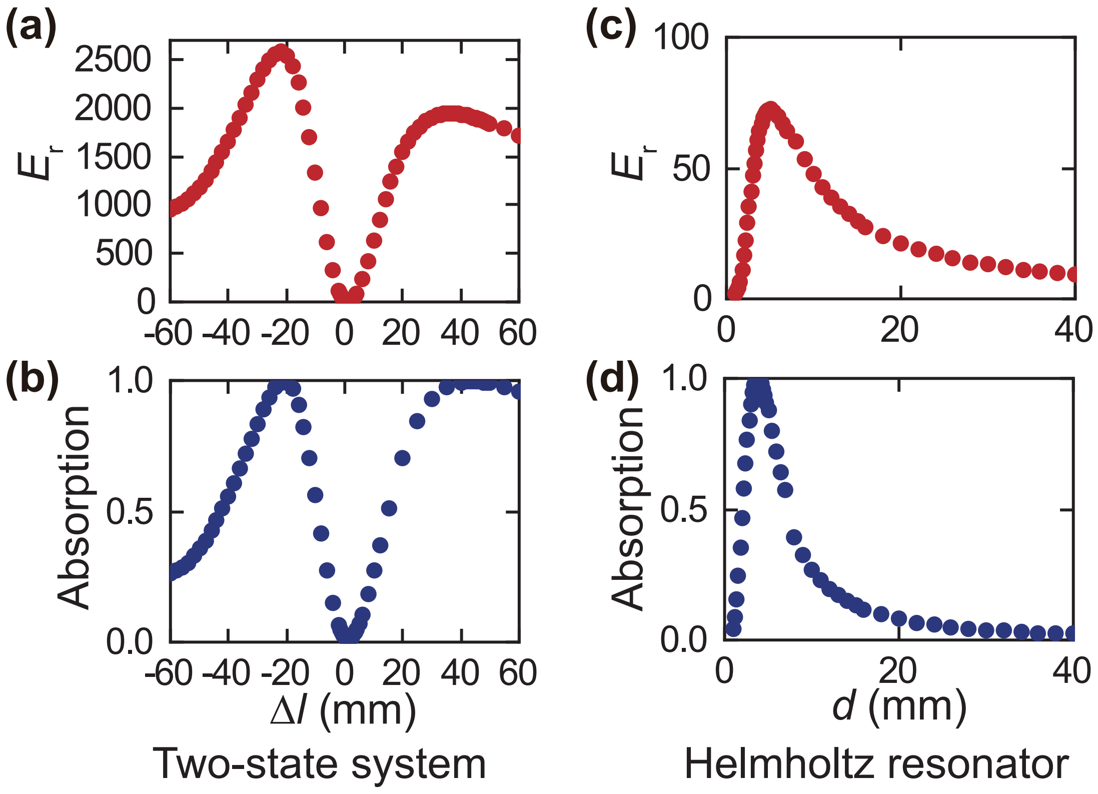

Figure S5. Absorption properties. a-b) Energy density enhancement and peak absorption coefficients of the two-state system in the presence of intrinsic loss. c-d) Energy density enhancement and peak absorption coefficients of the Helmholtz resonator in the presence of intrinsic loss.

Supplementary Text

1.7 Comparative analysis of open voltage and sound energy density amplification factor

Figure S6 shows the open voltage of the system (under 130 dB) and sound energy density amplification factor at the bottom of cavity B. The open voltage and the sound energy density amplification factor exhibit the same trend of variation. Notably, these two results have an identical peak frequency. The reason for the increase in sound energy density amplification factor indicates a gradual increase in the force exerted on the IXPP film. Consequently, the open voltage also rises correspondingly.

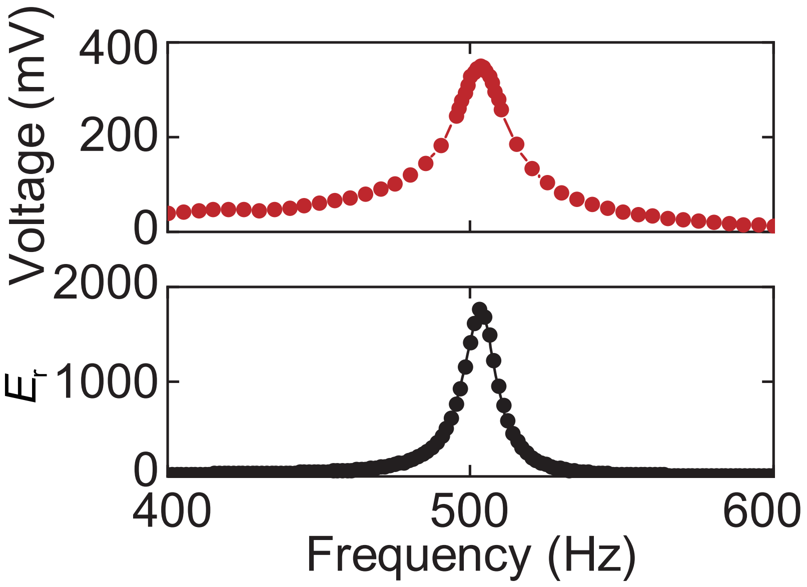

Figure S6. Open voltage properties. The experimental results of the open voltage of the system at an incident sound pressure of 130 dB (upper panel) and the sound energy density amplifications at the bottom of cavity B (lower panel).

Supplementary Text

1.8 Discussions on the future applications of self-powered acoustic sensors

Looking forward, self-powered acoustic sensors could find extensive applications. These include smart transportation systems where they can enhance operational efficiency and environmental monitoring. Moreover, their integration into wearable health devices and smart homes will improve personal and domestic well-being. These sensors are also ideal for

deployment in ocean monitoring and space exploration, where conventional energy sources are unfeasible. These capabilities will promote self-powered acoustic sensors to advance future sensing technologies and the Internet of Things.

Supplementary Text

1.9 Detailed information on the measured power

We have measured the electric power driving the loudspeaker, the acoustic power emitted from the loudspeaker, and the significantly enhanced local acoustic power on the IXPP in Figure 7. It can be observed from Figure S7c that at an incident sound pressure level of 130 dB, the electric power driving the loudspeaker is around 1000 mW, and the emitted acoustic power from the loudspeaker within the impedance tube is approximately 50 mW across each frequency. However, with the local field enhancement by the BIC-supporting system, the acoustic power at 501 Hz on the IXPP surface area can exceed 5000 mW. Besides, we have also measured the electric power used to light the LED, which is 4.3 mW. The electric power ($P = UI$) for both the loudspeaker and the LED was obtained by measuring the circuit current (I) and the voltage (U) across the device; acoustic power was calculated based on $W_{\mathrm{ap}} = S_i p_{\mathrm{a}}^2/(2\rho_0 c_0)$, where S_i is the calculation area of plane acoustic waves. Each experimental test was conducted with single-frequency acoustic waves, allowing the emitted sound pressure level to be modulated to target values using a LabVIEW program.

Supplementary Text

1.10 Discussions on the scenario of white noise incidence

When subjected to white noise, the presented BIC-supporting system will differentially amplify the pressure at various frequencies, enabling the IXPP to convert acoustic energy into electrical energy. Figure S7c-d illustrates the excitation acoustic power on the IXPP surface within the BIC-supporting system under the incidence of sound waves at the same acoustic power but at different frequencies. It is observed that the acoustic power at the BIC frequency is significantly higher than at other frequencies, resulting in the dominant influence of the acoustic waves near the BIC on the performance of the acoustic sensor. However, since white noise encompasses all frequencies, it fundamentally prevents the acoustic sensor from performing frequency-selective functions. Nonetheless, it is conceivable that this acoustic sensor can still be utilized to detect sound pressure level thresholds of white noise.

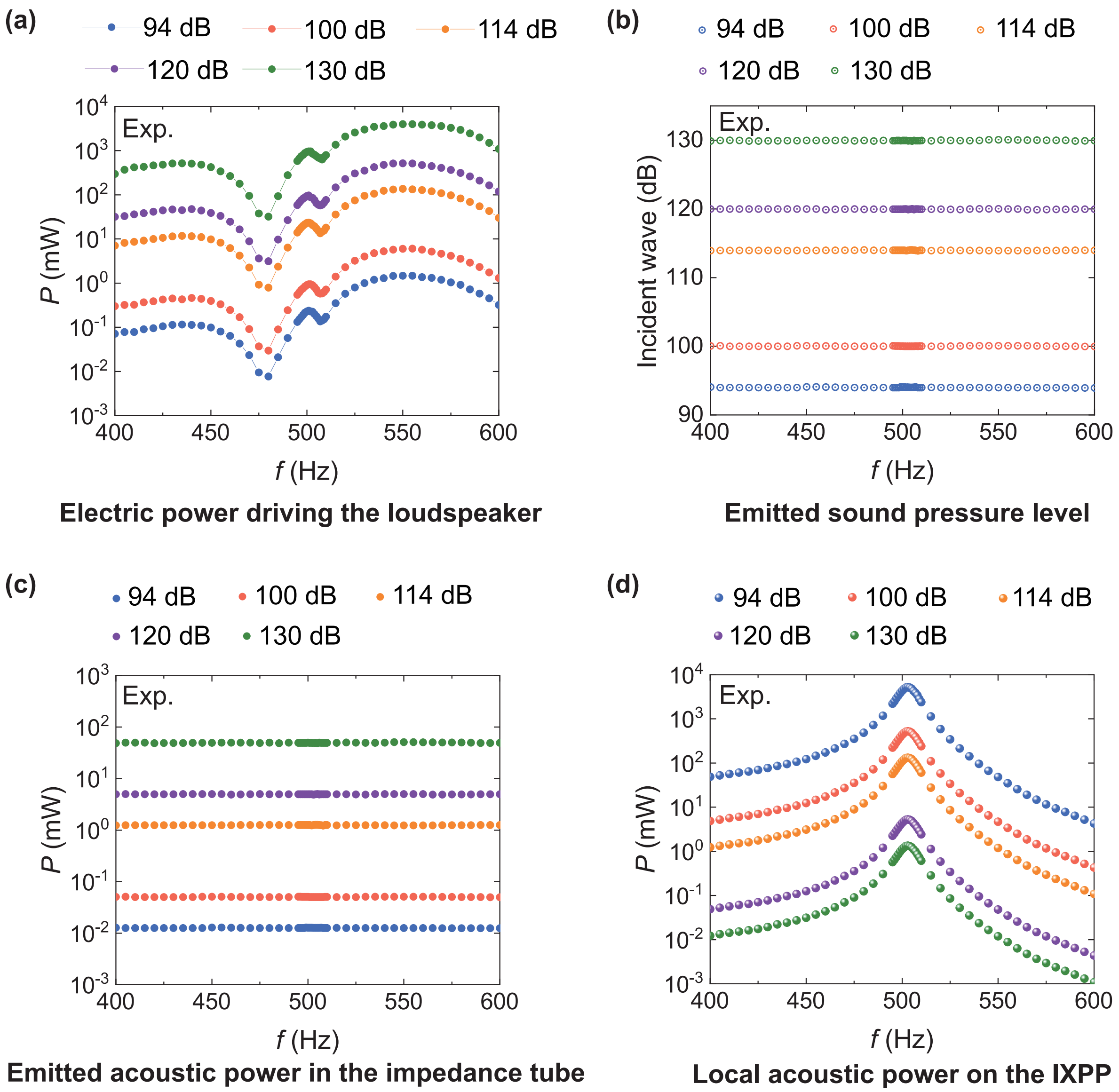

Figure S7. Measured electric power and acoustic power. a) Measured electric power driving the loudspeaker. b) Measured sound pressure level. c) Measured acoustic power emitted from the loudspeaker. d) Measured acoustic power on the IXPP surface area. Each experimental test was conducted with single-frequency acoustic waves, allowing the emitted sound pressure level to be modulated to target values using a LabVIEW program.

Movie S1

Movie S1 shows variations of the PCB board as the sound wave frequency changes between 490 and 510 Hz with 1 Hz intervals. The video shows a significant change in the brightness of the LED, which can be observed between 496 and 505 Hz.
